# The Role of the N-Terminal Domain of Thrombomodulin and the Potential of Recombinant Human Thrombomodulin as a Therapeutic Intervention for Shiga Toxin-Induced Hemolytic-Uremic Syndrome

**DOI:** 10.3390/toxins16090409

**Published:** 2024-09-20

**Authors:** Sarah Kröller, Jana Schober, Nadine Krieg, Sophie Dennhardt, Wiebke Pirschel, Michael Kiehntopf, Edward M. Conway, Sina M. Coldewey

**Affiliations:** 1 Department of Anesthesiology and Intensive Care Medicine, Jena University Hospital, 07747 Jena, Germany; sarah.kroeller@med.uni-jena.de (S.K.);; 2 Septomics Research Center, Jena University Hospital, 07745 Jena, Germany; 3 Department of Clinical Chemistry and Laboratory Medicine, Jena University Hospital, 07747 Jena, Germany; 4 Centre for Blood Research, Life Sciences Institute, Department of Medicine, University of British Columbia, Vancouver, BC V6T 1Z3, Canada; 5 Center for Sepsis Control and Care (CSCC), Jena University Hospital, 07747 Jena, Germany

**Keywords:** hemolytic-uremic syndrome, Shiga toxin, thrombomodulin, kidney injury, experimental HUS model, recombinant human thrombomodulin

## Abstract

Hemolytic-uremic syndrome (HUS) is a rare complication of an infection with Shiga toxin (Stx)-producing *Escherichia coli* (STEC-HUS), characterized by severe acute kidney injury, thrombocytopenia and microangiopathic hemolytic anemia, and specific therapy is still lacking. Thrombomodulin (TM) is a multi-domain transmembrane endothelial cell protein and its N-terminal domain has been implicated in the pathophysiology of some cases of HUS. Indeed, the administration of recombinant human TM (rhTM) may have efficacy in HUS. We used a Stx-based murine model of HUS to characterize the role of the N-terminal domain of TM. We show that mice lacking that domain (TMLed (−/−)) are more sensitive to Stx, with enhanced HUS progression seen at 4 days and increased mortality at 7 days post-HUS induction. In spite of these changes, renal function was less affected in surviving Stx-challenged TMLed (−/−) mice compared to their wild-type counterparts TMLed (+/+) at 7 days. Contrary to few clinical case reports from Japan, the administration of rhTM (0.06 mg/kg) to wild-type mice (C57BL/6J) with HUS did not protect against disease progression. This overall promising, but also contradictory body of evidence, requires further systematic preclinical and clinical investigations to clarify the role of TM in HUS as a potential therapeutic strategy.

## 1. Introduction

Infections with Shiga toxin (Stx)-producing *Escherichia coli* (STEC) can cause severe and bloody diarrhea, mostly affecting children under the age of five years. In up to 25% of these patients, Stx can trigger the thrombotic microangiopathy (TMA) hemolytic-uremic syndrome (HUS). HUS is characterized by a triad of acute kidney injury (AKI), thrombocytopenia, and microangiopathic hemolytic anemia. STEC is ingested as a contaminant of food, e.g., undercooked meat and vegetables, or water. STEC then colonizes the epithelial cells of the gut, after which Stx is released (as reviewed in [1]) and finds its way to multiple organs, particularly the kidneys [2]. Although the mechanisms are complex, briefly, Stx binds to the Stx-receptor globotriaosylceramide (Gb3) that is highly expressed by renal endothelial cells. The internalized Stx inhibits the target cell’s ribosomal apparatus and blocks protein biosynthesis, causing cell death. This, in turn, triggers the activation of a cascade of processes and signaling events that result in vascular dysfunction and renal injury, thrombus formation, hemolysis, and an inflammatory response with the invasion of immune cells into the renal tissue. Patients affected by STEC-associated HUS are currently managed with supportive measures, including isotonic volume expansion and, if required, renal replacement therapy. Specific therapeutic options are still lacking.

Thrombomodulin (TM; also known as CD141) is a single-chain type I transmembrane glycoprotein, predominantly expressed on the endothelium of blood and lymphatic vessels but also found in monocytes [3] and neutrophils [4] and in a soluble form in the blood [5]. TM is composed of five distinct domains, including an N-terminal domain (comprising a C-type lectin-like domain and a short hydrophobic region), epidermal growth factor (EGF)-like repeats, a serine-threonine rich domain, a single transmembrane domain, and a cytoplasmic tail ([6] and reviewed in [7]). The N-terminal domain is involved in inflammation, cell adhesion, and proliferation [8], while the EGF-like repeats bind to thrombin [9,10], where upon thrombin–TM activates protein C [11] and thrombin activatable fibrinolysis inhibitor (TAFI), thereby inhibiting coagulation and fibrinolysis [12].

Several in vitro and in vivo studies have implicated TM in the pathogenesis of HUS. Using a murine model of HUS triggered by the co-injection of Stx and lipopolysaccharide (LPS) as part of the outer membrane of Gram-negative bacteria, mice exhibited the excess activation of complement in conjunction with a reduced expression of TM by the glomerular endothelium [13], both of which were directly correlated with the severity of disease consistent with HUS. In a 48 h model of HUS, mice that lack the N-terminal lectin-like domain of TM (TMLed (−/−)) exhibited shortened survival, worse thrombocytopenia and renal dysfunction, and a more prominent immune response, with enhanced renal immune cell infiltration and chemokine upregulation [14].

By virtue of its anticoagulant properties, due to the inhibition of factors VIIIa and Va, the recombinant human soluble TM (rhTM), representing the entire extracellular domains of the molecule, has shown efficacy in treating disseminated intravascular coagulation (DIC). Indeed, rhTM is clinically approved for this purpose in Japan [15]. The use of recombinant soluble TM in patients with HUS is limited, and only a few case reports provide some evidence of its benefits [16,17], supporting the medical need for further study.

In this study, we analyzed the role of the N-terminal lectin-like domain of TM in a murine model of HUS [18], evaluating outcomes at days 4 and 7, comparing mice lacking the lectin-like domain ((TMLed (−/−)) with their wild-type counterparts. Using the information of these experiments, we additionally analyzed the effects of systemically treating wild-type mice with rhTM in the same model of HUS. In this study, we found that the N-terminal lectin-like domain of TM contributes in an early phase to the pathophysiology of HUS. However, we were not able to detect a therapeutic benefit of rhTM as a treatment in for HUS.

## 2. Results

### 2.1. Rate of HUS Progression Is Increased in TMLed (−/−) Stx Mice

**Figure 1:**
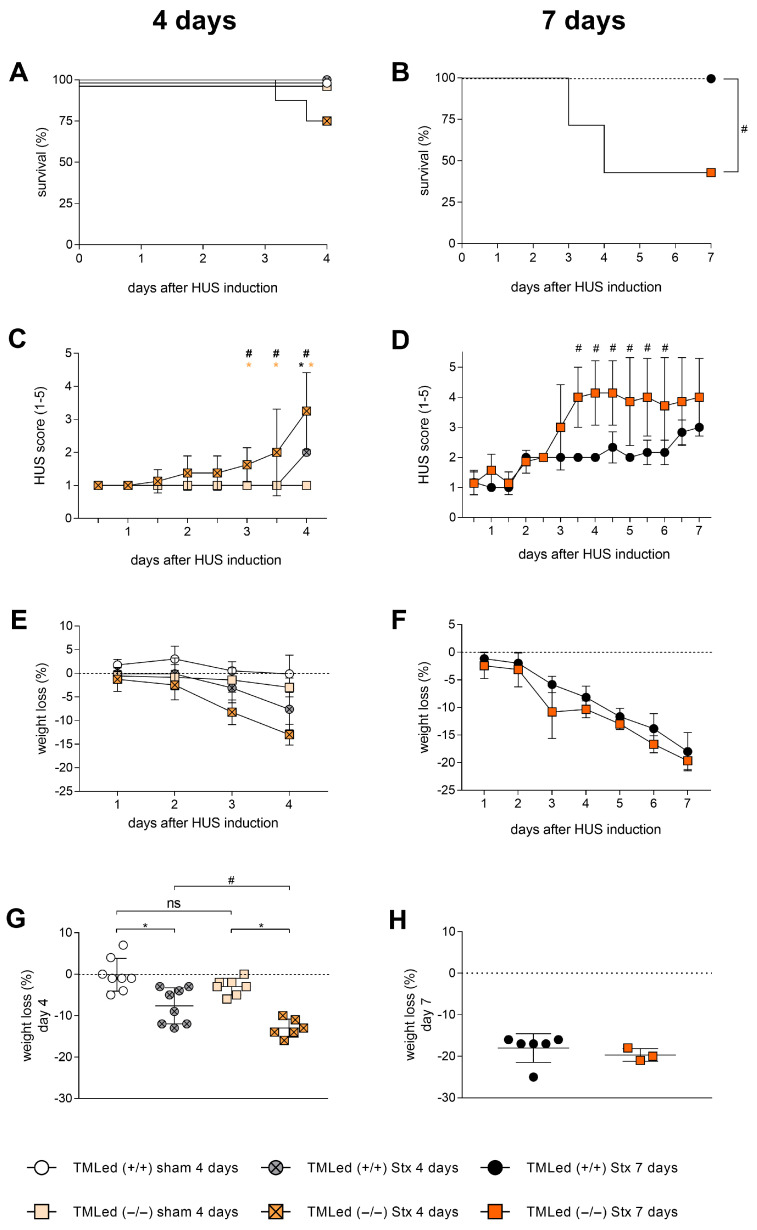
Disease progression of TMLed (+/+) and TMLed (−/−) mice with HUS at day 4 and day 7. Survival was followed up for (**A**) 4 days or (**B**) 7 days in sham and Stx-challenged mice at days 0, 3, and 6. Displayed are (**A**,**B**) survival; (**C**,**D**) analysis of HUS progression indicated by HUS score over the duration of experiment (ranging from 1 = no signs of illness to 5 = dead); (**E**,**F**) progression of weight loss over the duration of experiment; and (**G**,**H**) weight loss at the end of the experiment. (**A**,**C**): *n* = 7 for TMLed (−/−) sham; *n* = 8 for other groups; (**B**,**D**): *n* = 6 for TMLed (+/+) Stx and *n* = 7 for TMLed (−/−); (**E**–**H**): *n* = 3–8 mice per group (only surviving mice on day 4 or day 7). Data are presented with mean + SD. (**A**,**B**) Survival by Kaplan-Meier survival analysis + post hoc test. (**C**,**D**) Two-way-ANOVA with Tukey’s multiple comparisons test. (**G**) One-way ANOVA + Sidak’s multiple comparisons test and (**H**) Mann-Whitney test. * *p* < 0.05 vs. corresponding sham group; # *p* < 0.05 TMLed (+/+) Stx vs. TMLed (−/−) Stx. HUS, hemolytic-uremic syndrome; Stx, Shiga toxin; ns, not significant.

In the 4-day and 7-day HUS experiments, the survival of TMLed (−/−) Stx mice was decreased after exposure to Stx (Figure 1A,B), but only with statistical significance at 7-day experiment. Disease progression, as measured by our previously reported HUS score [19], was significantly worse in TMLed (−/−) Stx mice compared to TMLed (+/+) Stx mice (Figure 1C) by 3 days after HUS induction. The starting point and the rate of increase in the HUS score was faster in the TMLed (−/−) Stx mice in the 7-day experiments (Figure 1D) starting at 3.5 days after HUS induction. Interestingly, HUS scores in both genotype mice were not significantly different 7 days after the initial Stx exposure (Figure 1D). There was no statistical difference in the weight of sham TMLed (+/+) and TMLed (−/−) mice, excluding the possibility that TMLed (−/−) mice are genetically predisposed.

All Stx-challenged mice of both genotypes lost weight during the course of the 4- or 7-day HUS experiments (Figure 1E,F). However, at day 4, TMLed (−/−) Stx mice lost significantly more weight compared to the TMLed (+/+) Stx mice (Figure 1G). By day 7, this statistical difference was no longer observed, possibly suggesting some degree of slowdown by that time point (Figure 1H).

### 2.2. Renal Injury Is Less Pronounced in TMLed (−/−) Stx Mice at Day 7

**Figure 2:**
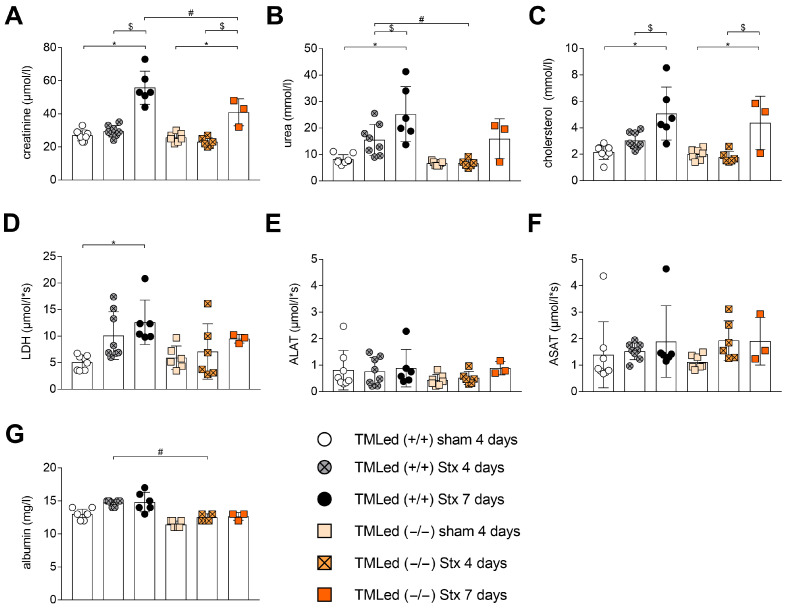
Parameters of renal injury, hemolysis, and liver injury of TMLed (+/+) and TMLed (−/−) mice with HUS at day 4 and day 7. Determination of plasma (**A**) creatinine, (**B**) urea, (**C**) cholesterol, (**D**) LDH activity, (**E**) ALAT, (**F**) ASAT, and (**G**) albumin. (**A**–**C**,**E**) One-way ANOVA + Sidak’s multiple comparisons test. (**D**,**F**,**G**) Kruskal–Wallis test + Dunn’s multiple comparisons test. *n* = 3–8 per group (only surviving mice with blood withdrawal on day 4 or day 7). Data are presented with mean + SD. * *p* < 0.05 vs. corresponding sham group. $ *p* < 0.05 Stx 4 days vs. Stx 7 days (same genotype). # *p* < 0.05 TMLed (+/+) Stx vs. TMLed (−/−) Stx. Stx, Shiga toxin; LDH, lactate dehydrogenase; ALAT, alanine transaminase; ASAT, aspartate transaminase.

At day 4, plasma creatinine levels as a marker for kidney dysfunction were measured. Plasma creatinine levels remained normal in TMLed (+/+) Stx or TMLed (−/−) Stx mice, similar to their corresponding sham mice (Figure 2A). At day 7, however, plasma creatinine levels significantly increased in both the TMLed (+/+) and the TMLed (−/−) Stx groups compared to their corresponding sham-treated groups. Notably, however, at that time point, plasma creatinine was significantly lower in the TMLed (−/−) Stx mice compared to the TMLed (+/+) Stx mice. Moreover, with the TMLed (+/+) Stx mice, plasma urea levels increased at day 4 and remained significantly elevated at day 7 compared to their corresponding sham group (Figure 2B). In contrast, TMLed (−/−) Stx mice plasma urea concentrations did not significantly rise on either day 4 or day 7. Day 4 plasma urea levels were significantly lower in the TMLed (−/−) Stx mice compared to the TMLed (+/+) Stx mice. Plasma cholesterol, as a marker of chronic renal insufficiency (reviewed in [20]), was not altered from normal levels at day 4 for the Stx mice of both genotypes (Figure 2C). However, it was significantly increased at day 7 for the Stx mice of both genotypes compared to their sham groups and their day 4 Stx groups (Figure 2C). As a measure of hemolysis and/or organ injury, we also measured the plasma LDH levels. These were increased in the TMLed (+/+) Stx mice on day 4 and significantly increased in TMLed (+/+) Stx mice at day 7 compared to their corresponding sham group (Figure 2D). Notably, LDH levels did not rise significantly in the TMLed (−/−) Stx mice. There was no evidence of liver injury, as evaluated by plasma ALAT and ASAT, in Stx mice of either genotype at day 4 or day 7 (Figure 2E,F). Plasma albumin levels were lower in the TMLed (−/−) mice compared to the TMLed (+/+) mice (Figure 2G) but not significantly different between the sham groups of either genotypes.

### 2.3. Altered Erythrocyte Characteristics and Immune Cell Populations in Whole Blood of TMLed (−/−) Stx Mice

**Figure 3:**
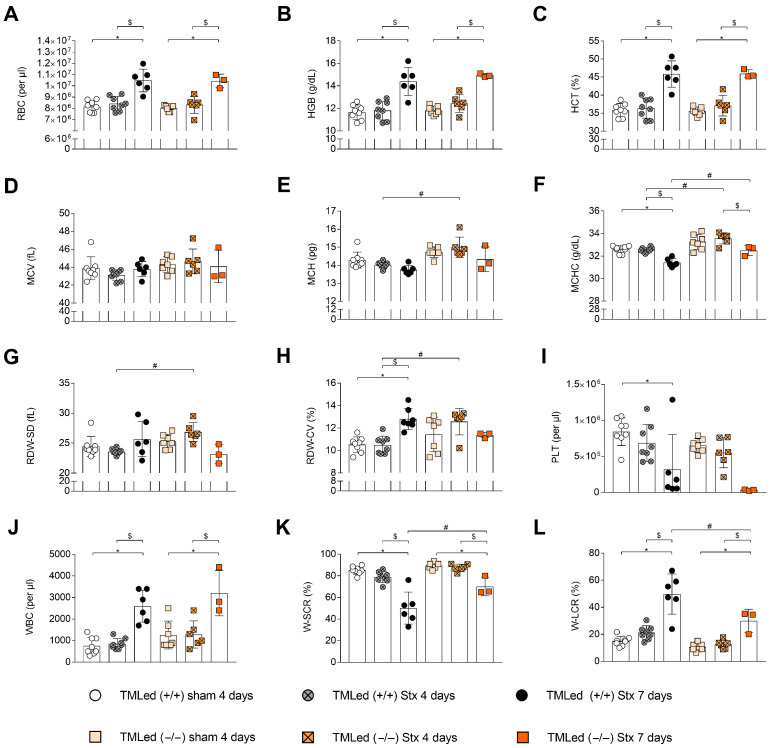
Hematological parameters of TMLed (+/+) and TMLed (−/−) mice with HUS at day 4 and day 7. Determination of whole blood (**A**) RBC, (**B**) HGB, (**C**) HCT, (**D**) MCV, (**E**) MCH, (**F**) MCHC, (**G**) RDW-SD, (**H**) RDW-CV, (**I**) PLT, (**J**) WBC, (**K**) W-SCR, and (**L**) W-LCR. (**A**–**C**,**F**,**J**–**L**) One-way ANOVA + Sidak’s multiple comparisons test. (**D**,**E**,**G**–**I**) Kruskal–Wallis test + Dunn’s multiple comparisons test. *n* = 3−8 per group (only surviving mice with blood withdrawal on day 4 or day 7). Data are presented with mean + SD. * *p* < 0.05 vs. corresponding sham group. $ *p* < 0.05 Stx 4 days vs. Stx 7 days (same genotype). # *p* < 0.05 TMLed (+/+) Stx vs. TMLed (−/−) Stx. Stx, Shiga toxin; RBC, red blood cell; HGB, hemoglobin; HCT, hematocrit; MCV, mean corpuscular volume, MCH, mean corpuscular hemoglobin; MCHC, mean corpuscular hemoglobin concentration; RDW, red blood cell distribution width; PLT, platelet; WBC, white blood cell; W-SCR, white cell–-small cell ratio (including lymphocytes); W-LCR, white cell- large cell ratio (including neutrophils).

At day 4 following initial exposure to Stx, erythrocyte concentrations (Figure 3A), hemoglobin (Figure 3B), and hematocrit (Figure 3C) were unaltered in the Stx mice of both genotypes. In contrast, the Stx groups of TMLed (+/+) as well as TMLed (−/−) displayed an increased concentration of erythrocytes, hemoglobin, and hematocrit at day 7, consistent with increased hemoconcentration during the course the disease, as previously described for this model of HUS [18]. The mean corpuscular volume (MCV) was unaltered in the Stx mice of both genotypes compared to their corresponding sham groups (Figure 3D) at day 4 and day 7. While there was no difference in the mean corpuscular hemoglobin (MCH; Figure 3E) between the TMLed (+/+) Stx mice and their corresponding sham group, TMLed (+/+) Stx mice had a lower mean corpuscular hemoglobin concentration (MCHC; Figure 3F) and increased red blood cell distribution width (RDW-CV; Figure 3H). There were no statistical differences in the levels of these parameters for TMLed (−/−) Stx mice at day 7 compared to their corresponding sham group. It is notable that TMLed (−/−) Stx mice showed significantly higher MCH (Figure 3E), MCHC (Figure 3F), RDW-SD (Figure 3G) and RDW-CV (Figure 3H) compared to TMLed (+/+) Stx mice at day 4. However, these differences seem to be, except for MCHC and RWD-CV, less prominent by day 7. During the course of the experiment, platelet counts decreased in the Stx groups of both genotypes at day 7 (Figure 3I). While the white blood cell counts in the Stx groups of both genotypes were significantly increased at day 7 (Figure 3J) compared to their corresponding sham groups and Stx groups at day 4, lymphocyte percentage was significantly increased in TMLed (−/−) Stx mice (Figure 3K), while neutrophil counts (Figure 3L) were significantly lower compared to TMLed (+/+) Stx mice at day 7.

### 2.4. Administration of rhTM Does Not Alter HUS Disease Progression in Wild-Type Mice

**Figure 4:**
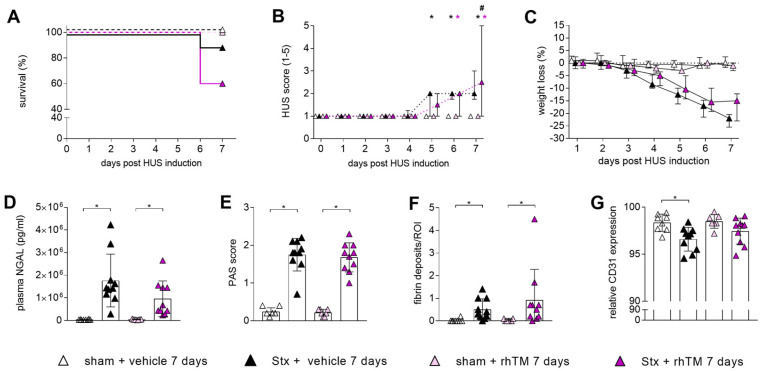
Clinical presentation and parameters of kidney injury of mice with HUS treated with rhTM. HUS was followed up for 7 days in sham and Stx-challenged mice with i.v. injection of rhTM (0.06 mg/kg bodyweight) on day 0, 3, and 6. (**A**) Survival is displayed by Kaplan–Meier survival analysis + post hoc test. (**B**) HUS progression is indicated by HUS score (ranging from 1 = no signs of illness to 5 = dead) and (**C**) progression of weight loss over the duration of experiment. (**D**) Plasma NGAL was determined on humane endpoint or day 7. Quantification of (**E**) PAS reaction, (**F**) fibrin deposits and (**G**) relative CD31 expression in renal sections at the end of the experiment (day 7 or humane endpoint). (**B**) Two-way-ANOVA with Tukey’s multiple comparisons test. (**D**,**G**) One-way ANOVA + Holm–Sidak’s multiple comparison test. (**E**,**F**) Kruskal–Wallis test + Dunn’s multiple comparison test. *n* = 8 for sham + vehicle; *n* = 10 for Stx + vehicle and Stx + rhTM; *n* = 7 for sham + rhTM. Data are presented with mean + SD. * *p* < 0.05 vs. corresponding sham group; # *p* < 0.05 Stx + vehicle vs. Stx + rhTM. HUS, hemolytic–uremic syndrome; Stx, Shiga toxin; rhTM, recombinant human thrombomodulin; NGAL, neutrophil gelatinase-associated lipocalin; PAS, periodic acid Schiff; CD31, cluster of differentiation 31.

We evaluated rhTM as a treatment for HUS using our Stx-based murine model. Following exposure to Stx, wild-type mice received three doses of rhTM over the course of 7 days. Survival was decreased, but not significantly altered, for Stx mice treated with rhTM (60%) and vehicle (90%) compared to their corresponding sham groups (Figure 4A). Over the 7-day experimental period, the Stx mice of both groups showed evidence of disease progression, based on the increasing HUS score (Figure 4B), beginning on day 5 for vehicle-treated Stx mice and on day 6 for rhTM-treated Stx mice. Moreover, on day 7, Stx mice treated with rhTM showed a significantly higher HUS score compared to Stx mice without treatment. Both groups of Stx mice showed in parallel a reduction in weight over the course of 7 days (Figure 4C). Stx mice treated with or without rhTM exhibited significantly increased levels of plasma NGAL compared to their corresponding sham groups (Figure 4D), indicating severe injury of the kidneys. Renal tissue injury was further evaluated by PAS staining. The Stx mice of both groups showed morphological changes indicated by a significantly increased PAS score compared to their corresponding sham groups (Figure 4E and representative images in Supplementary Figure S1A). AFOG staining was performed to identify fibrin deposition, which were significantly increased in both Stx groups compared to their corresponding sham group (Figure 4F and representative images in Supplementary Figure S1B). Only the vehicle-treated Stx mice exhibited a significantly lowered expression of CD31, an indicator of intact endothelial cells, whereas the rhTM-treated Stx mice showed a non-significant decrease in comparison to their corresponding sham group (Figure 4G and representative images in Supplementary Figure S1C).

### 2.5. Hematologic Parameters Are Not Significantly Affected by Administration of RhTM in Wild-Type Mice with HUS

**Figure 5:**
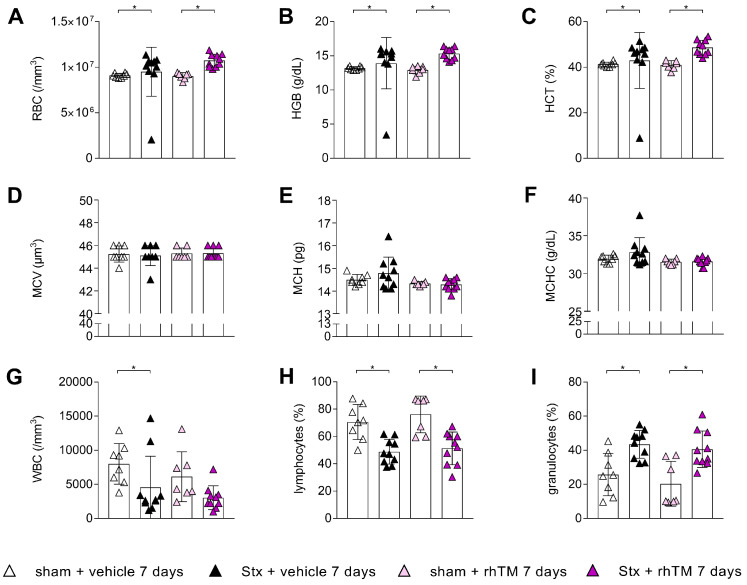
Hematological parameters of mice with HUS treated with rhTM. Determination of whole blood (**A**) RBC, (**B**) HGB, (**C**) HCT, (**D**) MCV, (**E**) MCH, (**F**) MCHC, (**G**) WBC, (**H**) lymphocytes, and (**I**) granulocytes on humane endpoint or day 7. (**A**–**D**,**F**,**H**,**I**) Kruskal–Wallis test + Dunn’s multiple comparison test and (**E**,**G**) one-way ANOVA + Holm–Sidak’s multiple comparison test. *n* = 8 for sham + vehicle; *n* = 10 for Stx + vehicle and Stx + rhTM; *n* = 7 for sham + rhTM. Data are presented as mean + SD. * *p* < 0.05. HUS, hemolytic–uremic syndrome; rhTM, recombinant human thrombomodulin; RBC, red blood cell; HGB, hemoglobin; HCT, hematocrit; MCV, mean corpuscular volume; MCH, mean corpuscular hemoglobin; MCHC, mean corpuscular hemoglobin concentration; WBC, white blood cells.

In whole-blood analyses, both groups, Stx-mice treated with rhTM or vehicle, showed a significant increase in the erythrocyte count (Figure 5A), hemoglobin (Figure 5B), and hematocrit (Figure 5C) compared to their corresponding sham groups, findings that are consistent with hemoconcentration, as previously noted [18]. Erythrocyte MCV (Figure 5D), MCH (Figure 5E), and MCHC (Figure 5F) showed no significant differences between the Stx groups and their corresponding sham groups. Vehicle-treated Stx mice had significantly decreased circulating leukocyte concentrations, whereas the rhTM-treated Stx mice only showed a non-significant decrease, compared to their corresponding sham groups (Figure 5G). Both Stx groups showed a significant decrease in lymphocytes (Figure 5H) and a significant increase in granulocytes (Figure 5I) compared to their corresponding sham groups.

## 3. Discussion

### 3.1. Lack of the N-Terminal Domain of TM in Mice Accelerates Early Disease Progression of Experimental HUS Apart from Kidney Injury

Transgenic mice lacking the N-terminal lectin-like domain of TM were previously reported to exhibit increased susceptibility to endotoxemia, with shortened survival and increased evidence of inflammation [21]. Furthermore, the N-terminal domain of TM was found to have a protective role for endothelial dysfunction in diabetic nephropathy [22,23]. These findings led to the hypothesis that the N-terminal domain of TM may also influence HUS progression since endothelial dysfunction and heightened immune responses participate in HUS pathology [24].

In the first part of our study, we analyzed the disease progression of TMLed (−/−) Stx mice compared to TMLed (+/+) Stx mice at 4 days and 7 days. Somewhat surprisingly, based on the HUS score and body weight at 4 days, disease progression was more rapid in TMLed (−/−) Stx mice. This genotype-dependent difference, however, appeared to be transient, as the HUS scores and weight loss were not different at day 7 in the two genotypes. We also observed a decrease in survival of the Stx TMLed (−/−) mice in the 4-day and 7-day experiments but recognize that our studies are limited by small sample size. Our findings of apparent increased severity of early disease progression in the TMLed (−/−) mice are, however, consistent with a previous report using a different HUS model (i.e., combination of Stx and LPS from *E.coli* O111:B4) in the same mice [14], supporting the notion that the N-terminal lectin-like domain of TM is likely involved in the early progression of HUS.

To evaluate the effect of the N-terminal domain on renal function in our HUS model, we measured plasma creatinine and urea at days 4 and 7. Interestingly, renal dysfunction was more pronounced in TMLed (+/+) Stx mice compared to the TMLed (−/−) Stx mice. This is in contrast to a previous report for experimental HUS triggered by Stx/LPS [14], where the blood urea nitrogen, also indicating kidney function, was significantly higher at day 1 in the Stx/LPS-treated TMLed (−/−) mice. In addition to this being a different model, in that report, the blood urea nitrogen was only measured at days 1 and 2 after the injection of high concentrations of Stx/LPS. Thus, comparisons may not be valid. To assess the influence of the N-terminal domain of TM in HUS, blood parameters were analyzed. The development of hemoconcentration, being a characteristic of the used model [18,19], was not different between the Stx groups of both genotypes. We did observe a transient effect on the erythrocyte characteristics in the TMLed (−/−) mice; however, the underlying mechanisms for this remain unclear. Additional analyses revealed that the total WBC count increased during HUS progression for both Stx groups at day 7, contributed primarily by an increase in the percentage of lymphocytes, with a decrease in the percentage of neutrophils in the TMLed (−/−) Stx mice at day 7. The small group sizes in our study, especially in TMLed (−/−) mice with HUS at 7 days, also needs to be considered. In future studies, verifying experiments for TMLed (−/−) Stx mice should be performed.

### 3.2. Therapeutic Intervention with RhTM Does Not Alter Disease Progression in Experimental HUS

Based on preclinical evidence, rhTM was developed as a drug for potential benefit in disorders associated with excess coagulation and inflammation [25]. Thus, in several clinical studies, rhTM has shown efficacy for patients with sepsis-induced disseminated intravascular coagulation (DIC) with AKI [26,27]. It has thus been approved for use in Japan for the treatment of DIC associated with malignant tumors or infectious diseases [28]. Its efficacy in the thrombotic microangiopathy of HUS [29] has only been evaluated to a limited extent [30].

Based on the data from TMLed (−/−) experiments in this study and others that states the lack of the N-terminal domain of TM has a negative impact on HUS, it has been hypothesized that rhTM as a therapeutic intervention would have beneficial effects in experimental HUS. Using our murine model of HUS, the administration of rhTM (0.06 mg/kg) did not yield an improvement in disease progression or renal injury. Indeed, there was a trend, albeit non-significant, of increased mortality in the rhTM-treated mice with HUS. Our findings are in contrast to those of Suyama et al. [30] who, using a more severe model of Stx/LPS-HUS, demonstrated a protective effect of rhTM, in terms of survival, renal function, and release of inflammatory cytokines [30]. These apparent discordant responses may be attributable to differences in the HUS models but also may reflect differences in the dose and type of the rhTM. The rhTM dosage in this study (0.06 mg/kg bodyweight) was used since this has indicated the therapeutic dosage for an rhTM protein used in different clinical studies (ART-123) [31,32,33]. However, the rhTM compound (ART-123) used in the Japanese studies and the rhTM compound used in our study contain the extracellular domain, including the N-terminal domain, the EGF-like domain, and the O-glycosylation mucin domain [31,34]. They differ, however, in length with 498 [31,34] and 491 (this study) amino acids, respectively. Since ART-123 is only available in Japan, the production and purification process could differ from the preparation used in this study.

A previous trial with rhTM for children with HUS has suggested that there may indeed be benefit of rhTM treatment in HUS [16]. However, such results must be interpreted with caution, as the intervention with the rhTM was started at different time points and other supportive measures were simultaneously administered. Nevertheless, it seems worthy to continue further study with different dosing schedules and in larger scales even though our apparently negative findings regarding the therapeutic utility of rhTM in Stx-HUS.

## 4. Conclusions and Outlooks

In summary, the results of this study lead us to the conclusion that the N-terminal lectin-like domain of TM contributes to the pathophysiology of HUS in a murine model of this disease and verifies the results of other pre-clinical studies. We were able to show that, in the early phase of HUS, the lack of the N-terminal domain of TM portends a more rapid early progression of disease and increased mortality at 7 days. While this study did not uncover a therapeutic benefit of rhTM in our experimental model of HUS, further larger-scale studies are required to more confidently define the role of this intervention.

## 5. Materials and Methods

### 5.1. TMLed (−/−) and TMLed (+/+)–Study Design in Stx-Based Model of HUS

Male TMLed (−/−) and TMLed (+/+) mice (age 8–9 weeks) were generated from Swiss/129/sv/ev embryonic stem cells and were backcrossed to C57BL/6J background. Mice were bred in the animal facility of university hospital Jena and were housed under standardized laboratory conditions, receiving standard rodent chow and water ad libitum. Stx2 was isolated (in-house manufacture), as described previously [18], from an O157:H7 EHEC strain 86–24, originating from a patients isolate. Briefly, after the 7-day acclimatization phase, murine HUS was induced by i.v. injections of 25 ng/kg bodyweight Stx doses (<0.1 EU/mL endotoxin), while sham mice were subjected to 0.9% NaCl. For 4-day experiments, mice were subjected to Stx or 0.9% NaCl on days 0 and 3 (total of two injections), while for 7-day analysis, mice were subjected to Stx or 0.9% NaCl on days 0, 3, and 6 (total of three injections). Mice were randomly assigned to sham or Stx groups: TMLed (−/−) sham (*n* = 7); TMLed (−/−) Stx 4 days (*n* = 8); TMLed (−/−) Stx 7 days (*n* = 6); TMLed (+/+) sham (*n* = 8); TMLed (+/+) Stx 4 days (*n* = 8); and TMLed (+/+) Stx 7 days (*n* = 7). Mice were identified by tail marking. Additionally, all mice received 800 µL of Ringer’s lactate solution as fluid resuscitation s.c. three times daily. Body weight was measured every 24 h, and HUS score, as described below, was evaluated blindly three times daily. Survival was assessed up to day 4 (4-day experiment) or day 7 (7-day experiment), or mice were sacrificed when reaching humane endpoints. At the end of the experiment, mice were sacrificed by exsanguination under deep anesthesia, induced with ketamine (100 mg/kg bodyweight) and xylazine (10 mg/kg bodyweight). All animal experiments were performed in accordance with the German legislation. Authorization was granted by the regional animal welfare committee (Thuringia State Office for Food Safety and Consumer Protection, Bad Langensalza, Germany) under the registration number 02-058-14.

### 5.2. Intervention with rhTM–Study Design in Stx-Based Model of HUS

Male C57BL/6J mice (age 12–14 weeks) were randomly assigned to the following groups: sham + vehicle; Stx + vehicle; sham + rhTM; and Stx + rhTM. Mice were bred in the animal facility of the university hospital Jena and were housed under standardized laboratory conditions, receiving standard rodent chow and water ad libitum. Stx2 was isolated (in-house manufacture), as described previously [18], from an O157:H7 EHEC strain 86–24, originating from a patient’s isolate. After the 7-day acclimatization phase, murine HUS was induced by three i.v. injections of 25 ng/kg bodyweight Stx doses (<0.1 EU/mL endotoxin) on days 0, 3, and 6, while sham mice were subjected to 0.9% NaCl. Mice were randomly assigned to the following groups: sham + vehicle 7 days (*n* = 8); Stx + vehicle 7 days (*n* = 10); vehicle + rhTM 7 days (*n* = 8); and Stx + rhTM 7 days (*n* = 10). For sample size calculation a priori, G*Power 3 was used as described by Faul et al., 2007 [35]. Mice received 0.06 mg/kg bodyweight rhTM (<1 EU/µg endotoxin; PeproTech, Cranbury, NJ, USA) 1 h after the Stx/NaCl i.v. injections on day 0, 3, and 6. Additionally, mice received 800 µL of Ringer’s lactate solution as fluid resuscitation s.c. three times daily. Body weight was measured every 24 h, and HUS score, as described below, was evaluated blindly three times daily. Survival was monitored up to day 7, or mice were sacrificed when reaching humane endpoints. Mice were sacrificed by exsanguination under deep anesthesia, induced with ketamine (100 mg/kg bodyweight) and xylazine (10 mg/kg bodyweight). All animal experiments were performed in accordance with the German legislation and approved by the regional animal welfare committee (Thuringia State Office for Food Safety and Consumer Protection, Bad Langensalza, Germany) under the registration number UKJ-18-019. One mouse (group: sham + rhTM day 7) was excluded from all analysis due to kidney cysts (*n* = 7 for all analyses).

### 5.3. HUS Score

HUS score was used as described previously (Supplementary Material [18,19]) to evaluate disease progression. Briefly, the score is based on a range of criteria, including activity, reaction, posture, general symptoms (weight loss), neurological symptoms, and fur quality. The score was calculated from the sum of all criteria, leading to score (1): no signs of illness; score (2): low-grade signs of illness; score (3): mid-grade signs of illness; score (4): high-grade signs of illness; and score (5): dead. A detailed point system is described in Supplementary Material of [19] and was applied in this study.

### 5.4. Blood and Plasma Analysis and Histological Stainings

For TMLed (−/−) and TMLed (+/+) mice experiments, blood withdrawal was performed, wherever possible, and hemograms were determined using the pocH100iV system (Sysmex, Kobe, Japan) as described previously [18]. Subsequently, plasma albumin, cholesterol, creatinine, lactate dehydrogenase (LDH) activity, urea, ASAT, and ALAT were measured using an Architect c16200/ci8200 automated clinical chemistry system (Abbott Diagnostics, Abbott Park, IL, USA) as described previously [18]. For day 4 and day 7 comparison, only mice that survived the planned endpoint were included. For rhTM administration mice experiments, the determination of hemograms was performed using scil Vet abc Plus+ (scil animal care company GmbH, Viernheim, Germany). Plasma neutrophil gelatinase-associated lipocalin (NGAL) was determined using LEGEND MAX^TM^ Mouse NGAL ELISA (Biolegend, San Diego, CA, USA) according to the manufacturer’s instructions. Organ preparation and histological stainings for the evaluation of kidney injury (PAS), endothelial damage (CD31) and thrombus formation (AFOG) were performed as described previously [19,36].

### 5.5. Statistics

All values are depicted as mean + SD for *n* number of animals tested. Statistical analyses were performed with GraphPad 7.03 (GraphPad Software, San Diego, CA, USA). Normality was tested using the Shapiro–Wilk normality test with a confidence level of 95%. For analysis of HUS score over the course of the experiment, two-way-ANOVA with Tukey’s multiple comparisons test was used. Non-parametric data were analyzed using Kruskal–Wallis test and Dunn’s multiple comparisons test, while one-way ANOVA and Holm–Sidak’s multiple comparisons test was used for parametric data. For the verification of Gaussian distribution, the Shapiro–Wilk normality test at 0.05 significance level was used. If necessary, values were converted to logarithmic values and tested again for Gaussian distribution to achieve normality. The corresponding sham groups were compared to 4-day and 7-day experiment (same genotype) as well as 4 days and 7 days experiments (same genotype). Moreover, day-4 or day-7 experiments were compared between both genotypes. Comparison between two groups was performed using the Mann–Whitney test. A *p*-value of <0.05 was considered as significant.

## Data Availability

The raw data supporting the conclusions of this article will be made available by the authors upon request.

## Supplementary Materials

The following supporting information can be downloaded at: https://www.mdpi.com/article/10.3390/toxins16090409/s1, Figure S1: PAS, AFOG and CD31 stainings of mice with HUS treated with rhTM.
