# Supplementary material for: The Role of the N-Terminal Domain of Thrombomodulin and the Potential of Recombinant Human Thrombomodulin as a Therapeutic Intervention for Shiga Toxin-Induced Hemolytic-Uremic Syndrome
Source: Toxins (Basel). 2024 Sep 20;16(9):409. doi: 10.3390/toxins16090409 (PMC11435709; doi:10.3390/toxins16090409)
Supplement: Supplementary file 1 [file toxins-16-00409-s001.zip › toxins-3195376-supplementary.pdf]

Supplementary Materials: The Role of the N-Terminal Domain of Thrombomodulin and the Potential of Recombinant Human Thrombomodulin as a Therapeutic Intervention for Shiga Toxin-Induced Hemolytic-Uremic Syndrome

Sarah Kröller, Jana Schober, Nadine Krieg, Sophie Dennhardt, Wiebke Pirschel, Michael Kiehntopf, Edward M. Conway and Sina M. Coldewey

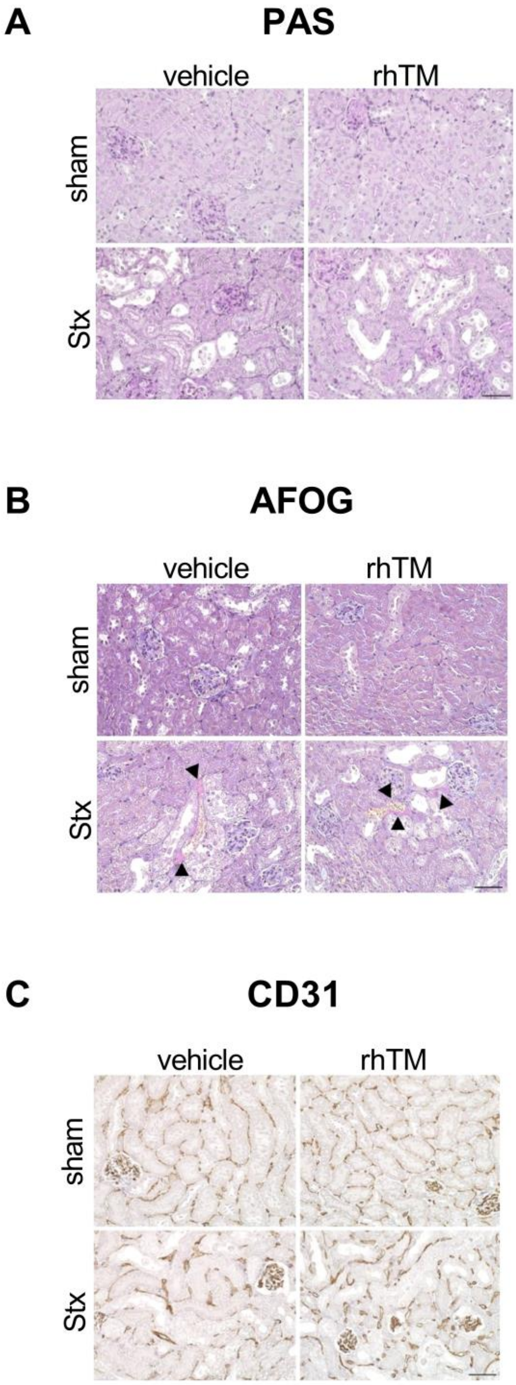

Figure S1. PAS, AFOG and CD31 stainings of mice with HUS treated with rhTM. Representative images of renal (A) PAS, (B) AFOG and (C) CD31 staining of sham mice and mice with HUS treated with rhTM on humane endpoint/ day 7. Bars = 50 µm (magnification 400x). Arrows indicate sites of fibrin deposition. Quantifications are shown in Figures 4E (PAS), 4F (AFOG) and 4G (CD31). HUS, hemolytic-uremic syndrome; Stx, Shiga toxin; rhTM, recombinant human thrombomodulin; PAS, periodic acid Schiff; CD31, Cluster of differentiation 31; AFOG, acid-fuchsin-orange-G
